# Magnitude and antimicrobial susceptibility profile of bacteria isolated from pediatric sepsis cases at University of Gondar Hospital, Northwest Ethiopia

**DOI:** 10.1186/s12887-024-04969-8

**Published:** 2024-08-01

**Authors:** Gashaw Amsalu, Feleke Moges, Geta Bayu, Baye Gelaw

**Affiliations:** 1 Department of Medical Microbiology, School of Biomedical and Laboratory Sciences, College of Medicine and Health Sciences, University of Gondar, Northwest, Ethiopia; 2 Department of Pediatrics and Child Health, School of Medicine, College of Medicine and Health Science, University of Gondar, Northwest, Ethiopia

**Keywords:** Septicemia, Bacteremia, Antimicrobial susceptibility, Blood samples, Children, pediatrics, Ethiopia

## Abstract

**Background:**

Sepsis is one of the major causes of morbidity and mortality among pediatric patients throughout the world. The varying microbiological pattern of sepsis warrants the need for researches on the causative organisms and their antimicrobial susceptibility pattern. The epidemiology of neonatal and pediatric sepsis in Ethiopia is under-research. The objective of this study was to evaluate the burden of bacterial pathogens and their antimicrobial susceptibility patterns among children suspected of sepsis.

**Methods:**

An institutional-based prospective cross-sectional study was conducted on 370 pediatric(age birth-15 years) patients suspected of sepsis at the University of Gondar Comprehensive Specialized hospital from December 2020 to November 2021. Blood samples were collected aseptically and inoculated into Tryptone Soya Broth for culture. The organisms grown were identified by standard microbiological methods and subjected to antibiotic susceptibility testing by modified Kirby-Bauer disk diffusion method recommended by Clinical laboratory and standard institute. Methicillin resistance was confirmed using Cefoxitin disk diffusion method. Data entry and analysis were done using Statistical Package for Social Sciences (SPSS) version 26 software. A p-value less than 0.05 at 95% confidence interval was considered statically significant.

**Results:**

Out of the total 370 study subjects, 21.6% (80/370) of them were culture positive. Of these, 43 (53.8%) and 37 (46.3%) were Gram-positive and Gram-negative bacterial pathogens, respectively. The most prevalent Gram-positive bacterial isolate was *Staphylococcus aureus* (*n* = 24; 30%) and coagulase negative staphylococci (*n* = 7; 8.8%). Among the Gram-negative bacterial isolates, the leading bacteria was *Klebsiella pneumoniae* (*n* = 20; 25%) followed by *Escherichia coli* (*n* = 7; 8.8%). Clindamycin, Chloramphenicol, Gentamicin and Ciprofloxacin were the most effective antibiotics against Gram-positive bacterial isolates while Amikacin, Meropenem and Chloramphenicol were effective against Gram-negative pathogens. Methicillin resistance was detected in 45.8% of *Staphylococcus aureus*. Multi-drug resistance (MDR) antimicrobial susceptibility pattern was observed in 76% of the bacterial isolates.

**Conclusion:**

Gram positive bacteria were the predominant isolates among pediatric sepsis cases and most of the bacterial isolates showed MDR. *Staphylococcus aureus* and *Klebsiella pneumoniae* were frequently isolated bacteria. The high prevalence of drug resistance warrants rational use of antibiotics and the need for regular antibiotic susceptibility surveillance studies.

## Introduction

Sepsis is a systemic inflammatory reaction syndrome that occurs because of a suspected or confirmed infection, commonly caused by bacterial pathogens [1]. Briefly, to be septic, a child must have a confirmed or suspected infection and signs of a systemic response to that infection. Sepsis among pediatrics remains a leading cause of morbidity and mortality and poses a major global public health challenge. An estimate from meta-analysis of population-based studies on neonatal and pediatric sepsis showed about 3.0 million cases of neonatal sepsis and 1.2 million cases of pediatric sepsis annually worldwide, highlighting the tremendous burden of sepsis as a critical outcome of infectious diseases [2]. Predeveloped countries with large populations of children bear the major burden of pediatric sepsis. Key contributing factors include poor sanitation, low birth weight, insufficient immunization and nutrition, lack of awareness about of sepsis, insufficient training for medical personnel, and limited access to reliable laboratory tests, especially blood cultures. Additionally, individuals living in remote areas face compromised care, outdated antibiotic guidelines, and the rise of drug-resistant infections [3]. Considering sub-Saharan African countries, septicemia remains a major health issue, responsible for 30–70% of illnesses and deaths among children [4, 5].

Resistance to commonly used antibiotics is a rapidly growing problem with significant health, economic, and social implications globally. This issue largely stems from the frequent use of antibiotics without proper bacteriological and drug susceptibility evidence [1]. Additionally, the rise of multidrug-resistant organisms further limits available antibiotic options [6]. Inadequate treatment of sepsis exacerbates the condition, leading to patient fatalities and the emergence of new drug-resistant strains [7].

The increasing frequency of antimicrobial resistance found in bloodstream infections is of great concern for African countries where access to care and broad-spectrum antimicrobials is often limited [8]. Different research works conducted in Ethiopia revealed that drug resistance is increasing to the commonly used antimicrobial agents [9]. The epidemiology of pathogens most often associated with sepsis and the emergence of drug resistant isolates varies with geographic location and time period; therefore, continuous surveillance is necessary in each region [10]. Continuous surveillance is important for the development of institutional guidelines based on local microbial prevalence and their antibiotic susceptibility patterns [10]. The World Health Organization (WHO) in its 2017 report, recognized the scarcity of epidemiological data on sepsis in general and pediatrics sepsis in particular particularly among the low and middle income countries (LMICs), and urged member States to take specific actions to reduce the burden of sepsis [11, 12]. In Ethiopia, available studies are largely focused on neonatal sepsis and few epidemiological studies addressed pediatric sepsis that made the essential information system available so deficient to implement appropriate control strategies. Therefore, the objective of the current study was to describe the etiologic agents and the rational use of antibiotics of pediatric sepsis at the University of Gondar Comprehensive Specialized Hospital.

## Materials and methods

### Study design and settings

This cross-sectional study was conducted at the University of Gondar Comprehensive Specialized Hospital (UoGCSH) from December 2020 to November 2021. UoGCSH, one of the largest teaching hospitals in the Amhara National Regional State, provides a wide range of services including surgical, medical, pediatric, gynecologic, obstetric, oncologic, and ophthalmologic care to a community of over seven million people from the Amhara, Tigray, and Benishangul Gumuz regions. As a multidisciplinary specialized hospital, it features 700 inpatient beds, an operating room, intensive care units, a fistula center, 13 different wards, and outpatient departments. The hospital provides services for more than 1.5 million people.

### Study population

The study participants were Pediatric patients aged birth to 15 years who visited the hospital during the study period. Children suspected of sepsis and visiting the emergency, inpatient and outpatient departments of the hospital were recruited and those Pediatric patients presenting with clinical features suggestive of sepsis were included in the study.

### Sample size determination

Single population proportion formula was used to calculate the sample size. The calculated sample size was 370. A simple random sampling technique was used and eligible patients during the study period were included in the study until the sample size was reached.

### Blood sample collection and laboratory procedures

Using aseptic techniques, cleaning the venous site with 70% alcohol and subsequently by 10% iodine solution, 2–5mL venous blood samples were drawn by experienced nurses and inoculated into culture bottles containing 10 ml tryptone soya broth. Blood culture broths were then transported within 30 min to the University of Gondar Compressive Specialized Hospital microbiology laboratory. All blood cultures were incubated at 37℃ under aerobic conditions for 24 h and culture broths were continuously inspected daily for 7 days until growth is detected.

### Bacterial identification

Blood culture bottles that showed evidence of bacterial growth such as turbidity and/or hemolysis were sub-culture on to Blood agar, Chocolate agar, MacConkey agar and Mannitol salt agar. Inoculated Blood agar, Mannitol salt agar and MacConkey agar plates were incubated at 37℃ overnight in aerobic incubator for incubation of the culture plate while Chocolate agar plates were maintained under increased CO_2_ tension using a candle jar to enhance growth of fastidious organisms.

Bacterial isolates were identified by colony morphology, Gram stain reaction, and Biochemical tests. Biochemical tests were performed from sub- cultured pure bacterial colonies. For Gram-positive bacteria, catalase, coagulase, novobiocin and mannitol fermentation were performed. For Gram-negative bacteria, triple sugar iron agar (TSI), citrate utilization test, urease test, motility, indole, H_2_S production and Lysine decarboxylase (LDC) tests were used and the standard operational procedure (SOP) of the University of Gondar Comprehensive specialized Referral Hospital were followed for bacterial identification.

### Antimicrobial susceptibility test

Once the bacteria is isolated and identified, the standard Kirby-Bauer disk diffusion method was used to determine the antimicrobial susceptibility profiles of the isolates. The Kirby–Bauer disk diffusion method was used with Mueller–Hinton agar to determine the antibiotic susceptibility patterns of the isolates, and CLSI M100 was used to interpret the results [13]. The antimicrobial susceptibility testing for Gram positive isolates was performed against: Penicillin (10U), Ampicillin (10 µg), Ceftriaxone (5 µg), Vancomycin (30 µg), Erythromycin (15 µg), Chloramphenicol (30), Gentamycin (10 µg), Ciprofloxacin (5 µg), Clindamycin (2 µg), Oxacillin (1 µg) Cefoxitin (30 µg). For Gram negative isolates: Gentamycin (10 µg), Ampicillin (10ug), Ceftriaxone (30 µg), Cefazolin(30 µg), Chloramphenicol (30 µg), Trimethoprim -sulphamethoxazole (Co-trimoxazole) (25 µg), Amoxicillin clavulanic acid (AUG) (30 µg), Cefepime(30 µg), Ceftazidime(30 µg), Ciprofloxacin (5 µg), Amikacin (30 ) and Meropenem (10 µg) disks were considered. Bacterial Isolates resistant to one or more antibiotic types in three or more antibiotic classes were considered multidrug-resistant [14].

### Isolation and identification of Methicillin resistant *Staphylococcus aureus*

*Staphylococcus aureus* bacterial isolates were sub-cultured on Mannitol Salt Agar (MSA) and tested by Catalase test, oxidase test, and coagulase test and those isolates positive for coagulase were further characterized. Methicillin resistant *S. aureus* (MRSA) was identified by using Cefoxitin disk. Plates containing the Cefoxitin disk were examined following a 24-hour incubation period at 35℃. The diameter of the zone of inhibition (ZOI) (< 21 mm) of the bacterial growth around the antibiotic disc was recorded. The clear zones (inhibition zones) of bacterial growth around the antibiotic disc (including the discs) diameter for individual antimicrobial agents were measured and then translated into sensitive (S) and resistant (R) categories according to the interpretation table of the Clinical and Laboratory Standard Institute (CLSI) [15].

### Data management and analysis

All the collected data were entered into Microsoft Excel Sheet and analyzed through Statistical Package for Social Sciences (SPSS), version 26. Accordingly, descriptive statistics such as percentages and frequency distribution were used to determine the prevalence. Frequencies and cross tabulations were used to summarize descriptive statistics. Descriptive statistics was also used to explain antimicrobial susceptibility patterns. A bivariate logistic regression was performed to show any association between independent variables and the outcome variable (sepsis). P value < 0.05 was considered as statistically significant.

### Operational definition:-

Sepsis is Systemic inflammatory response syndrome (SIRS) to infection. SIRS, defined by the presenceof ≥ 2of the following criteria (abnormal temperature or white cell count must be one of the criteria): Abnormal core temperature(< 36°Cor > 38.5 °C), abnormal heart rate, Raised respiratory, abnormal white cell count in circulating blood [1]. The most frequent of which is fever. This definition is typically for older children while infants and neonates usually present with non-specific symptoms [16].

## Results

### Age and sex wise distribution of study participants

A total of 370 blood samples from clinically suspected cases of pediatric sepsis patients were included. The proportion of males to females enrolled in the study were 199 (53.7%) and 171 (46.2%), respectively. Most of the participants (*n* = 287; 77.5%) were less than 5 years of age (Table 1).

**Table 1:** Sex and age distribution of sepsis suspected Pediatrics patients at the University of Gondar Comprehensive specialized hospital, Northwestern Ethiopia

Variables	Category	Total cases (*n* = 370)	Positive cases (*n* = 80(%)	OR (CI)	*P*-value
sex	Male	199	50(62.5)	0.634(0.382–1.054)	0.079
	Female	171	30(37.5)	R	
Age	< 28 days	138	37(46.2)	4.152( 1.203–14.334)	0.024
	< 1year	77	22(27.5)	4.535(1.261–16.302)	0.021
	1–5 years	72	13(16)	2.497(0.664–9.389)	0.176
	6–10 years	46	5(6)	1.382 (0.308–6.205)	0.673
	11–15 years	37	3(3.8)	R	

OR = Odds ratio; R = Reference

### Empirical antibiotics prescription practice

Data of the current study showed that significant number of the patients (about 40% of the participants) had the exposure to antibiotics and took empirically without considering blood culture results. Among the 148 patients who were treated with empiric antibiotics, 26(17.6%) had positive blood culture result. Ceftriaxone 86(58%) were among the most common empirically used antibiotics during the study followed by Gentamycin, Ampicillin, Cloxacillin each accounting for 20.9% (*n* = 31) and that of Vancomycin for 18.9% (*n* = 28).

### Clinical features

Among the clinical features, the most common clinical finding was fever (*n* = 295; 79%) followed by increased respiratory rate (tachypnea) (*n* = 154; 41.6%) and hypothermia (*n* = 58; 15.7%). More than one clinical characteristics of sepsis were observed among two-thirds of the study subjects. When these characteristics were analyzed for their association to sepsis using bivariate analysis with backward logistic regression, hypothermia (OR = 2.203; 95%CI = 0.123–0.832 and *P* = 0.018) was found to be significantly associated with positive blood culture results (Table 2).

**Table 2:** Clinical characteristics of sepsis suspected pediatrics patients at the university of Gondar comprehensive specialized hospital, Northwestern Ethiopia

Clinical characteristics	Total *n* (%)	Culture positive(*n* = 80)	Odds ratio(95%CI)	*P*-value
Fever	293(79)	66(82.5)	1.282(0.675–2.435)	0.448
Hypothermia	56(15)	8(10)	2.203(0.123–0.832)	0.018
Increased respiratory rate	154(42)	32(40)	1.044(0.630–1.732)	0.867
Infective endocarditis	20(5.4)	6(7.5)	1.486(0.557-3.964)	0.428
Pneumonia	25(6.7)	2(2.5)	0.711(0.236–2.142)	0.544
Jaundice	15(4)	1(1.25)	0.316(0.0328–3.561)	0.335
seizures	9(2.4)	1(1.25)	0.445(0.0531–4.672)	0.743

### Bacterial profile

Out of the total 370 blood samples drawn from sepsis suspected pediatric patients, the frequency of culture positive specimens was 21.6% (*n* = 80). Only single bacterial species was isolated from each child (no mixed bacterial infection was detected). Among the culture positive cases, the proportion of male children were 62.5% (*n* = 50) and that of females 37.5% (30). Although the majority of the culture positive samples were from males, no statically significant gender differences were detected. Neonate (OR = 4.152; CI (1.203–14.334) *p* = 0.024) and children under one years of age groups had a statistically significant association to sepsis (OR = 4.533; CI(1.261–16.032) *p* = 0.021) (Table 1).

In this study, Gram-positive bacteria accounted for 53.8% (*n* = 43) and Gram-negative bacteria for 46.3% (*n* = 37). *Staphylococcus aureus* was the leading gram-positive isolate (*n* = 24; 30%) followed by Coagulase negative staphylococci (CONS) (*n* = 7; 8.8%). On the other hand, there were two *Streptococcus agalactiae* and one *Streptococcus pyogenes* bacterial isolates detected (Table 3).

**Table 3:** Frequencies of Gram negative and Gram positive bacterial pathogens isolated from blood cultures of sepsis suspected Pediatrics patients at the University of Gondar Comprehensive specialized hospital, Northwestern Ethiopia (December 2020 to November 2021)

Gram stain	Bacterial isolate	Frequency (%)
Gram + ve	*S. .aureus*	24 (30)
*n* = 43(53.8%)	*CoNS*	7 (8.8)
	*Enterococcus spp*	5 (6.3)
	*S.viridians*	4(5)
	*S.agalacia*	2(2.5)
	*S.pyogens*	1(1.3)
Gram -ve *n* = 37(46.3%)	*K.Pnuemoniae*	20(25)
	*E. coli*	7(8.8)
	*K.ozane*	3(3.8)
	*Citrobacter spp*	2(2.5)
	*Enterobacter spp*	2(2.5)
	*Pseudomonas spp*	2(2.5)
	*Acinetobacter spp*	1(1.3)
Total		80(100)

Among the *S. aureus* isolates (*n* = 24), 11(45.8%) of them were found to have MRSA. Among Gram negative bacteria, the leading bacteria identified was *K. pneumonia* with an isolation rate of 25% (*n* = 20) followed by *E. coli* 8.8% (*n* = 7). Others detected in low rate were *Klebsiella ozane*, Citrobacter specious, Enterobacter specious and Pseudomonas Spp. One Acinetobacter specious was also identified (Table 3).

### Antimicrobial sensitivity pattern of bacterial isolates

The antimicrobial sensitivity patterns of bacterial isolates of the current study showed that 76% were resistant to three or more different classes of antibiotics. The most common Multi drug resistance bacterial pathogens isolated in this study were *K. pneumoniae*, 83% followed by *E. coli*, 80% and *S. aureus*, 45%.

Data on the antimicrobial resistance pattern of specific antimicrobials showed that most of the Gram-positive bacteria were resistant to Penicillin (59%), Ampicillin (56%) and Erythromycin (50%). On the other hand, Gram-positive bacteria showed better susceptibility patterns for Vancomycin, Ciprofloxacin, Gentamycin, Clindamycin and Chloramphenicol. *S. aureus* isolates showed high resistance to Penicillin (62.5%) followed by Ampicillin (58%), Erythromycin (54%), Cefoxitin (45.8%), Ceftriaxone (41.6%) and Tetracycline (37.5%). Data also showed that *S. aureus* isolates drug resistance pattern was observed for Clindamycin (12.5%), Ciprofloxacin (25%), Gentamycin (29%) and Chloramphenicol (33%) (Table 4). On the other hand, 45.8% of the *S. aureus* bacterial isolates showed resistance to Methicillin and grouped as MRSA.

**Table 4:** Bacterial isolates and their antimicrobial sensitivity pattern of Gram-positive bacteria isolated from sepsis suspected children at the University of Gondar Comprehensive Specialized Hospital (December 2020 to November 2021)

Bacterial isolate	Resistant pattern (%)											
	*P*	AMP	OXA	CXT	CTR	E	TE	DA	CAF	CN	CIP	VAN
*S. aureus* (*n* = 24)	15(62.5)	14(58)	12(50)	11(45.8)	10(41.6)	13(54)	9(37.5) (37.5)	3(12.5)	8(33)	7(29)	6(25)	ND
CONS(*n* = 7)	5(71.4)	4 (57)	4(57)	3(42.8)	3(42.8)	4(57)	2(28.5)	1(14)	2(28.5)	1(14)	2(28.5)	1(14)
Enterococcus SPP(*n* = 5)	4(80)	3(60)	ND	ND	2(40)	4(80)	4(80)	1(20%)	0(0)	1(20%)	1(20)	1(20)

Keys : ND not detected, CoNS, Coagulase negative Staphylococcus, P penicillin, Amp Ampicillin, OXA Oxacillin, CXT cefoxitin; CTR Ceftriaxone, E Erythromycin, TE Tetracycline, DA Clindamycin, CAF Chloramphenicol, CN Gentamicin and CIP Ciprofloxacin, VAN vancomycin

Most of the tested antibiotics were not effective against Gram-negative bacteria isolates; resistant to Amoxicillin-clavulanate (89.2%), Ceftriaxone (88.9%), and Ampicillin (87%). However, Chloramphenicol and Ciprofloxacin showed more effectiveness against Gram-negative bacteria and almost all isolates were sensitive to Amikacin and Meropenem. *K. pneumoniae*, the predominant isolate among GNB, showed high resistance to Ampicillin, Ceftriaxone, Cefazolin and Cefepime (95% each) and Amoxicillin-clavulanate, Ceftazidime and Cotrimoxazole (90.5% each), Gentamicin and Ciprofloxacin (75% each). *E. coli* showed 100% resistance to Ampicillin and Amoxicillin-clavulanate and a marked resistance to Cefepime, Ceftriaxone, Ceftazidime and Cefazolin (85.7% each account). *E. coli* demonstrated a 28.5% resistance to Chloramphenicol and 100% sensitive to Meropenem and Amikacin (Table 5).

**Table 5:** Bacterial isolates and their antimicrobial sensitivity pattern of Gram negative bacteria isolated from sepsis suspected children at the University of Gondar Comprehensive Specialized Hospital (December, 2020 to November, 2021)

Bacterial isolate	Resistant pattern *n* (%)											
	AMP	AMC	CIP	CN	CZ	CTR	CAZ	CAF	SXT	CEP	AK	MER
*K.pneumoniae* (*n* = 20)	19(95)	18(90)	15(75)	15(75)	19(95)	19(95)	18(90)	8(40%	18(90)	19(95)	0(0))	2(10)
*E.Coli*(*n* = 7)	7(100)	7(100)	4(57)	4(57)	6(85.7)	6(85.7)	5(71.4)	2(28.5)	3(42.8)	6(85.7)	0(0)	0(0)

Amp = Ampicillin, AMC = Amoxicillin-clavulanate, CIP = Ciprofloxacin, CN = Gentamicin, CZ = Cefazolin, CTR = Ceftriaxone, CAZ = Ceftazidime, CAF = Chloramphenicol, SXT = Trimethoprime-Salfamethoprime (Co-trimoxazole ), CEP = Cefepime, AK = Amikacin, MER = Meropenem

## Discussion

Pediatric sepsis contributes substantially to children morbidity and mortality and is a major global public health problem. The detection, identification, and susceptibility testing of causative agents are essential for the proper management of the patient. Unfortunately, information regarding the spectrum of pathogens causing infection and their antibiotic susceptibility in many resource limited countries is limited [17]. Besides, sepsis among children was poorly investigated and this study provides information about the common etiologic agents and their antimicrobial susceptibility pattern.

In the present study 370 pediatric patients from birth to 15 years of age with clinical signs and symptoms of sepsis were included. The most prevalent clinical signs were fever (79%) followed by increased respiratory rate (tachypnea) (42%). However, hypothermia was the only statistically significant indicator of sepsis in the present study (*P* = 0.019). The encountering tachypnea and fever as predominating feature of sepsis is somehow consistent with previous report from Addis Ababa, Ethiopia [18].

The overall prevalence of culture positive bacteria caused pediatric sepsis was 21.6% (80/370). The blood culture positivity rate (21.6%) detected in this study is higher than what has been reported in Nepal (10.6%), Iran (9.1%) and Kenya (6.4%) [19–21]. However, it is lower than blood culture yields reported in Tanzania (29.8%), in Sub-Sahara African Setting (26.9%) and previous studies conducted in Ethiopia (29.8%) [18, 22, 23]. The lower prevalence in our study could be many of the study participants (40%) received empirical treatment with antibiotics before blood was drawn for culture. Moreover, the difference in the prevalence of pediatric sepsis might be associated with varying levels of infection control methods and the nature of participants. On the other hand, although not statistically significant, higher rate of sepsis were observed among males (62.5%) when compared with females (37.5%). Participants in under one year of age group including the neonates were the highest group infected (73.8%) compared with other age groups. This was supported by study report from USA and Nigeria [24, 25]. The immature immunity among neonates makes them more vulnerable to infection. Moreover, bodies of infants are less colonized by normal microbiota as the presence of well-established resident normal microbiota has a significant role in protection from pathogenic bacteria [26].

Gram positive bacteria (GPB) were more frequently isolated than Gram negative bacteria (GNB) in the present study. This agrees with most studies [8, 9, 27, 28]. However, study reports from Uganda and Addis Ababa showed high Gram-negative bacteria among children with sepsis [18, 29, 30]. The difference, the predominance of Gram-positive bacteria, might be related to the difference in the practice of using medical devices for which some Gram positive bacteria are directly associated or due to geographical variations or change in time. *S. aureus* and *K. pneumoniae* were the predominant bacterial isolates identified in the current study. Previous studies showed that *S. aureus* and *K. pneumoniae* isolates were also the major pathogens from children with sepsis (27–30%) [31–33]. This may be due to the fact that the ubiquitous nature of *S. aureus*, which is frequently found on the skin, and main cause of various type of infection in human beings [34]. *K. pneumoniae* is a normal member of gastro-intestinal flora and recently, has emerged as a significant cause of hospital acquired infections (septicemia and other types of infection). Among the Gram-positive bacteria, coagulase negative Staphylococcus (CoNS) were the second most common isolated (8.8%) following *S. aureus*. Detecting this bacterium as a second most prevalent isolate was also reported from Addis Ababa and Tanzania [18, 23]. Coagulase negative staphylococcus is part of normal flora and have long been considered as contaminants and were rarely reported to cause severe infections. However, these bacteria were reported to have emerged as a major cause of nosocomial infections and isolation of CoNS in children who are immune-compromised and those on advanced medical care such as mechanical ventilation and central line catheterization [35, 36]. The interpretation of their presence is a major concern for clinicians and clinical microbiology laboratories. The decision for therapy relies mostly on the observation of sepsis symptoms and the number of positive blood cultures. However, the criteria of multiple blood cultures could not be applied in this study in early age pediatric patients who could not undergo multiple venipuncture [19].

In this study, multi-drug resistance (MDR) was observed in the majority of the isolates (76%), with *K. pneumoniae*, *E. coli*, and *S. aureus* being the primary MDR bacterial strains. Previous studies have also reported high levels of multidrug resistance in bacteria isolated from blood cultures [27, 37]. Gram-negative bacteria are increasingly resistant to antibiotics that were previously effective. This is consistent with findings from other previously made studies showing significant resistance to these drugs [40]. The contributing factors for the high rate of antimicrobial resistance (AMR) among Gram-negative bacteria may be the overuse of antibiotics as empiric treatment which promotes the emergence of new resistance genes. This new resistant gene can be easily transferred among Gram-negative bacteria (GNB) through mobile genetic elements [41]. For example the inter-genus transfer of plasmid carried resistance genes for extended-spectrum beta-lactamases was observed between *E. coli* and *K. pneumoniae*, as well as among other organisms in the Enterobacteriaceae family [42]. Data from the current study showed that 90% of the gram negative bacteria were resistant to ceftriaxone. Third-generation cephalosporins, such as Cefotaxime, Ceftriaxone, and Ceftazidime, are frequently used when resistance to first-line antibiotics is suspected [43]. However, there was a notable increase in resistance to ceftriaxone, reaching 28% in this study compared to previous studies that reported resistance rates up to 62.5% [9, 27]. This increase is possibly due to the over-prescription of ceftriaxone, as more than 40% of the participants used the drug prior to blood culture. On the other hand, resistance to Amikacin and Carbapenems was low in this study, aligning with a meta-analysis from East Africa that estimated resistance of Klebsiella spp. to Amikacin and Carbapenems to be 5% and 0% [44].

## Conclusion

In conclusion, the present study showed that blood stream infection constituted about 21.6%. Gram positive bacteria were the predominant etiologic agents. Staphylococcus *aureus*, CONS, *K. pneumoniae and E. coli* were the commonest causative agents. The majority of the bacterial isolates showed MDR and this limits therapeutic options which warrant rational use of antibiotics and the need for regular antibiotic susceptibility surveillance studies.

## Data Availability

The data is available with the corresponding author, and it is possible to get upon request.

## Abbreviations

CONS: Coagulase negative staphylococci
GNB: Gram negative bacteria
GPB: Gram positive bacteria
LDC: Lysine decarboxylase
LMICs: Low and middle income countries
MDR: Multi-drug resistance
MRSA: Methicillin resistant S. aureus
SOP: Standard operational procedure
SPSS: Statistical Package for Social Sciences
TSI: Triple sugar iron agar
UoGCSH: University of Gondar Compressive Specialized Hospital
WHO: World Health Organization
ZOI: Zone of inhibition
